# VIAMD: a Software for Visual Interactive Analysis of Molecular Dynamics

**DOI:** 10.1021/acs.jcim.3c01033

**Published:** 2023-11-27

**Authors:** Robin Skånberg, Ingrid Hotz, Anders Ynnerman, Mathieu Linares

**Affiliations:** Linköping University, SE-581 83 Linköping, Sweden

## Abstract

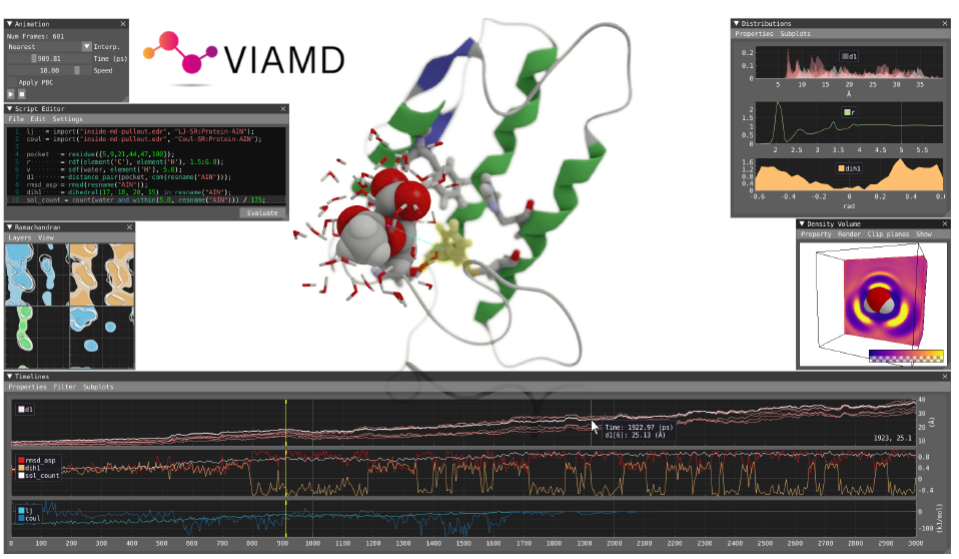

The typical workflow in molecular dynamics (MD) analysis requires several separate tools, often resulting in a lack of synergy and interaction between the individual analysis steps. This article presents VIAMD, an application designed to address this issue by integrating a 3D visualization of molecular trajectories with flexible analysis components. VIAMD uses an interactive scripting interface, allowing for property definition and evaluation. The application provides context-aware suggestions and expression feedback through information and visualizations. The user-defined properties can be explored and analyzed through the various components. This enables correlation with spatial conformations, statistical analysis of distributions, and powerful aggregation of multidimensional properties such as spatial distribution functions. VIAMD has the potential to advance research in many scientific disciplines and is a promising solution for improving the workflow of MD visualization and analysis.

## Introduction

Molecular dynamics (MD) analysis can be summarized as the process of studying properties from simulated molecular systems. During the simulation, a base set of properties, such as coordinates and energies, is periodically sampled and written as output. These base properties are then examined directly or used indirectly as inputs to derive new properties. Because MD is used in various scientific disciplines to study processes at the molecular level, there is natural variation in the properties studied. This property variation has led to the development of MD analysis tools that allow researchers to construct new properties from the basis set, often through a scripting interface using expressions and operations. The usual MD analysis workflow is an iterative process in which the MD systems are visualized using a 3D visualization tool to gain a spatial and temporal understanding of the system and its structures. Properties are then defined and computed using a scripting tool, and their values are plotted and studied. The plotted values can then be correlated with spatial and temporal geometric configurations displayed in the visualization tool, inspiring ideas for new hypotheses to test. While most molecular visualization tools facilitate some form of analysis, they are often task-specific and implemented as a separate plug-in. Task-specific means that it is designed to compute and plot a specific property, but its data are unavailable to other components in the application. When resorting to isolated tools in the analysis process, the potential synergy and interaction between the tools are left on the table. Tapping into this can potentially increase the efficiency and ease of use.

Molecular visualization tools, such as Chimera,^1^ VMD,^2^ Pymol,^3^ Caver,^4^ Jmol/Jsmol,^5^ Samson,^6^ Ovito,^7^ MegaMol,^8^ and Mol*,^9^ provide means of configuring visual representations, often with a focus on generating images for scientific publications. Many of the listed tools also provide some tools for analysis. Kozlikova et al.^10^ and Kut’ák et al.^11^ provide a comprehensive overview of the molecular visualization techniques used in the software mentioned above.

Property computation in the form of user-defined expressions through scripting is exposed through tools such as Collective Variables *Colvars*(12) and is available in software such as VMD,^2^ LAMMPS,^13^ NAMD,^14^ and GROMACS.^15^ MDAnalysis^16^ provides similar functionality but is implemented as a Python library. The analyzed properties are often time-varying scalar values derived from the atom coordinates of the trajectory, either as an aggregated measure of the complete trajectory or, more frequently, varying over trajectory frames.

More recently, Ulbrich et al.^17^ demonstrated the computation of user-defined properties through a visual node-based graph network instead of the typical scripting interface. A benefit of using visual nodes to construct expressions is the ability to visualize each node in the *expression tree*. sMolBoxes exploits this to plot intermediate results from the operations. The downside of visual node-based graph networks is the occupation of screen space, a limited resource. The graph can grow substantially for complex expressions, and the layout of nodes then has to be carefully considered not to end up with a spaghetti graph.

In this Article, we present VIAMD—software for Visual Interactive Analysis of Molecular Dynamics that builds upon an initial prototype^18^ created in Inviwo.^19^ The software has since then evolved and been redesigned to be applicable in a broader setting. The software is designed to optimize the workflow common in MD analysis by tightly linking the three main analysis tools: molecular visualization, property calculation, and property plotting. By placing the tools in the same application, we can leverage emergent synergies formed by tight coupling. Examples of such synergies include the ability to click and inspect points in time displayed by plots, enabling correlation of the value and conformation of structures. Another example is providing suggestions for properties from the user’s current selection and supporting the user in declaring properties by providing direct visual feedback asserting the operation and structures involved. The article introduces the software VIAMD by first providing a brief overview of the application, followed by individual sections that describe the components in more detail.

## Overview

**Figure 1:**
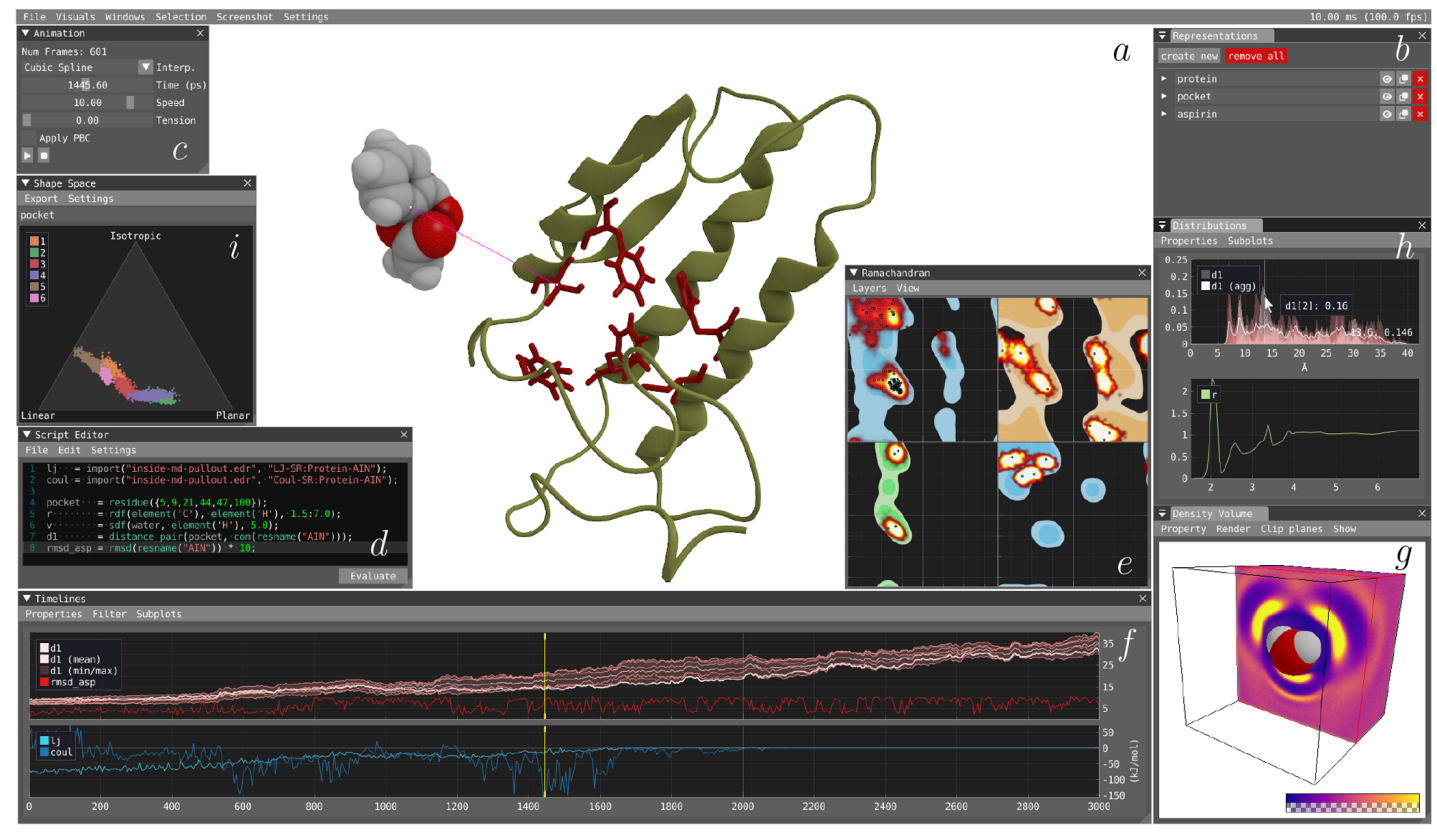
Overview of the application running with its components: spatial view (a), representation window (b), animation window (c), script editor (d), Ramachandran plot (e), temporal window (f), density volume window (g), distribution window (h), and shape space window (i).

The application is built around a 3D visualization of the molecular data set referred to as the spatial view, Figure 1a. In conjunction with the spatial view, several components are exposed, most of which are dedicated to analysis (Figure 1e,f,g,h,i). The central component orchestrating the analysis is the script editor, Figure 1d and Figure 3, where the user can write expressions and assign them to properties. The script is then evaluated over the trajectory frames, similar to *Colvars*. The user-defined properties form the core analysis entities of the application, and upon evaluation, they can be inspected in the various components of the application.

## Components of VIAMD

### Spatial View (a)

The spatial view, Figure 1a, provides a 3D view of the molecular system together with graphical primitives, which enhance and emphasize structures and operations within the system. The camera can be manipulated by clicking and dragging the mouse, and upon double-clicking on any geometry in the scene, the camera will use that point in space as its pivot point. The user can select atoms with the mouse by holding the shift key, where the left mouse button appends and the right mouse button removes items from the current selection. The user defines the system’s visual representations in the representations window, Figure 1b. Each representation is defined by a type, color map, and textual filter that defines the visible set of atoms.

### Selection and Interaction

**Figure 2:**
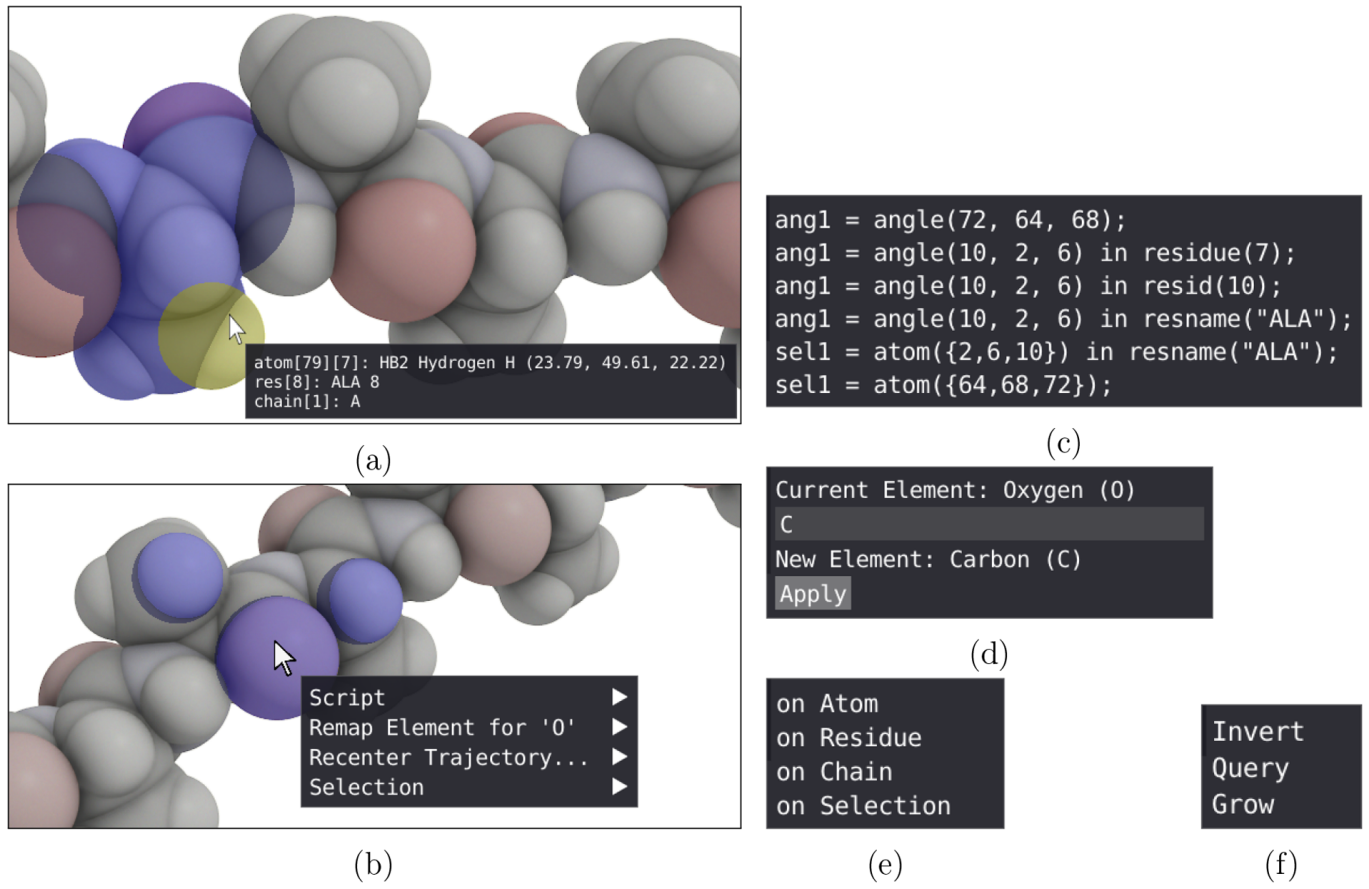
Selection vs highlight (a): Atoms part of the active selection are shown in blue, and the hovered atom is highlighted in yellow with an information window. The Spatial Context menu (b) exposes operations through submenus that can be applied to the data set. Script (c) supplies script suggestions based on active selection. Remap Element (d) provides a mechanism for remapping the assigned atomic element for specific labels. Recenter Trajectory (e) provides the option of recentering the trajectory, and Selection (f) exposes tools for manipulating the active selection.

The application exposes different ways of selecting and interacting with the data through different views, but all operate on a shared set of atoms representing *active* selection. If atoms are present in the active selection, the colors of the shown representations are desaturated and the selected atoms are shown in blue (Figure 2a). Similar to the active selection, a highlighted set of atoms (shown in yellow) is used to communicate structures involved in operations. When the user right-clicks in the spatial view, a spatial context menu (Figure 2b) is accessed, which offers the user a set of operations based on active selection.

#### Script Suggestions

With an active selection present, the user is provided with suggestions of script snippets based on the active selection. There are two categories of suggestions given: operations and selections. The operations result in basic function calls that match the number of selected entities, given some permutations of the context in which they are applied. Selection suggestions are the textual equivalent of the active selection expressed in the scripting language under different permutations. The active selection contains three atoms in Figure 2c. Therefore, the generated suggestions assume that the user intends to compute an angle. In general, there are many ways of expressing the same operation. In this case, the selected atoms belong to the same residue; therefore, the user is given options to perform this operation within the context of the residue. Note that the indexing of atoms changes when applied within a context and is, in such cases, local to the context.

#### Element Remapping

Some topology file formats lack atom element data, and in such cases, the application attempts to infer it from other data available, mainly the atom label. This approach is based on heuristics and will fail on certain occasions. In such a case, the user can modify the mapped element of the atoms through the Remap Element menu shown in Figure 2d. This operation can be applied to a single atom or to all atoms with the same label.

#### Recentering

The application supports dynamic recentering of trajectories: by selecting an atom, residue, chain, or arbitrary set of atoms, the context menu option seen in Figure 2e becomes available. The operation uses the center of mass of the supplied set to recenter each trajectory frame.

#### Selection Growth

When an active selection is present, growing that selection becomes available. The operation is applied either as a flood fill across covalent bonds or as a radial growth based on the distance to any atom in the active set.^26^

#### Periodic Boundary Conditions

When the application loads a trajectory frame into memory, atoms within structures are optionally translated to prevent them from being split across the periodic boundaries by applying Deperiodization. Structures in this context are implicitly defined by the covalent bonds formed between atoms. Any atoms connected by covalent bonds will belong to the same structure. Deperiodization of a structure is achieved by computing its center of mass, then applying Periodic Boundary Conditions to the center of mass to ensure that it resides within the viewed period of the system. Then, each atom of the structure is translated to the same period as the center of mass.

### Animation Window (c)

### Script Language and Editor (d)

**Figure 3:**
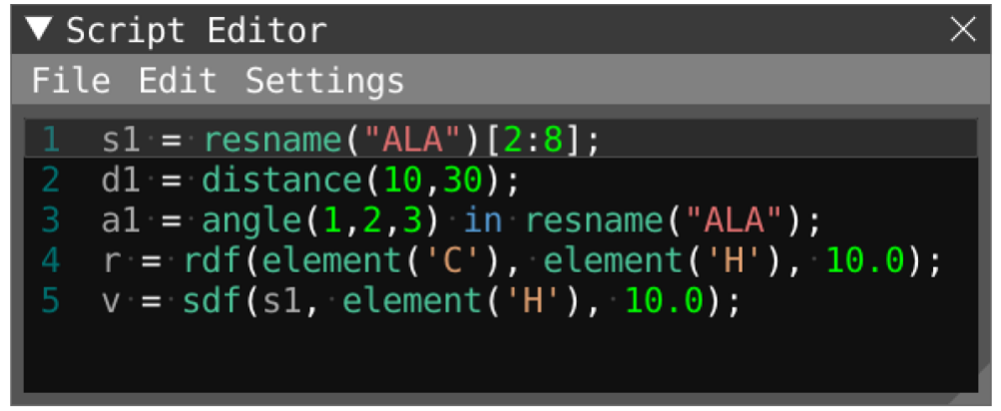
Script editor window: A text-based editor for defining properties and expressions evaluated for the loaded data set.


The scripting language used in the script editor (Figure 3) is a central part of VIAMD and has been designed from the ground up, focusing on MD analysis. It is used for both defining selections and expressing computational properties in the script editor. The motivation for designing a new language instead of choosing some existing high-level scripting language, e.g., Python, is two-fold. First and foremost, the language’s syntax can be simplified and task-focused, to support MD analysis. Second, it allows us to inspect and evaluate any part of an expression by traversing the abstract syntax tree. This grants the ability to provide feedback in the form of type information and to invoke visualizations of any part of any expression. The language is designed with declarative syntax, meaning the user declares the desired results rather than the more common imperative counterpart, where the user expresses the explicit algorithmic steps involved. This simplifies the syntax of the language and reduces its overall complexity. We refer the reader to the Supporting Information and online resources for listings of the available operations and more advanced examples.

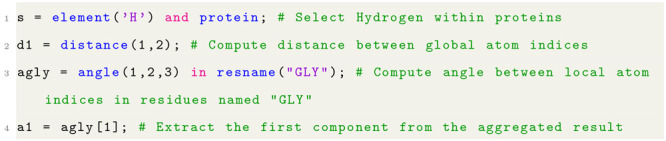
Listing 1: Example of the syntax of the VIAMD scripting language.

#### Variables and Properties

Variables are expressions assigned to identifiers that can then be referenced in other expressions. Variables of specific types (float[1..N], Distribution, and Volume) are promoted into Properties, which can be visualized in the various components of VIAMD. Float properties with a length greater than 1 do not hold a single value but a population of values. In the components of the application, they have the option of being shown as aggregates of the population. As examples, see Figure 1f,h and Figure 5 where user-defined properties d1 and a1 are shown as population aggregates, respectively. In the temporal window (f), aggregate properties can be shown individually or as population, min, max, mean, and variance. While in the distribution window (h), the aggregate populations can be shown individually or as an aggregate.

#### Selections

Selections are the results of filtering operations and are represented by sets of atoms. An example of a filtering operation resulting in a selection can be seen in line 1 in listing 1. The expression contains an *and* operation representing the intersection of subsets: *element*(′*H*′) and *protein*, where *element*(′*H*′) is the set of atoms with the element hydrogen and *protein* represents the set of atoms belonging to residues identified as proteins.

#### Contexts

Filtering operations are not limited to producing a single set of atoms but can produce multiple sets. For example, line 3 in listing 1, *resname*(*“GLY”*), results in multiple sets of atoms, each set representing a residue. The motivation is to allow the sets to serve as contexts for operations to be applied within them. This is referred to as contextual operations. Contextual operations are achieved by using the keyword *in*, where the left-hand side of the keyword expresses the operation to be performed and the right-hand side provides the contexts for the operation. Again, consider line 3 in listing 1, where the property *agly* is declared as the result of a contextual operation where the operation is to compute the angle between atoms 1, 2, and 3, and the contexts are each residue named *GLY*. In isolation, the angle operation yields a single scalar value, but since this operation is evaluated within multiple contexts, the resulting type will have a length matching the number of evaluated contexts.


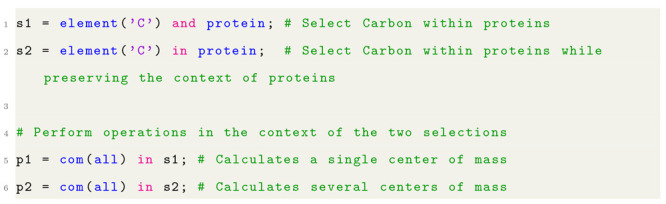
Listing 2: Example of *and* vs *in* keywords and how they differ.

At first glance, the keywords seem to offer similar functionality. However, the difference is that *in* preserves contexts while *and* implicitly flattens the result into a single set. Consider listing 2, where two selections are created, s1 and s2, each representing the same set of atoms. The key difference is that s2 maintains the contexts of the proteins, and subsequent operations that use the selections may differ in their results.

#### Script Editor

**Figure 4:**
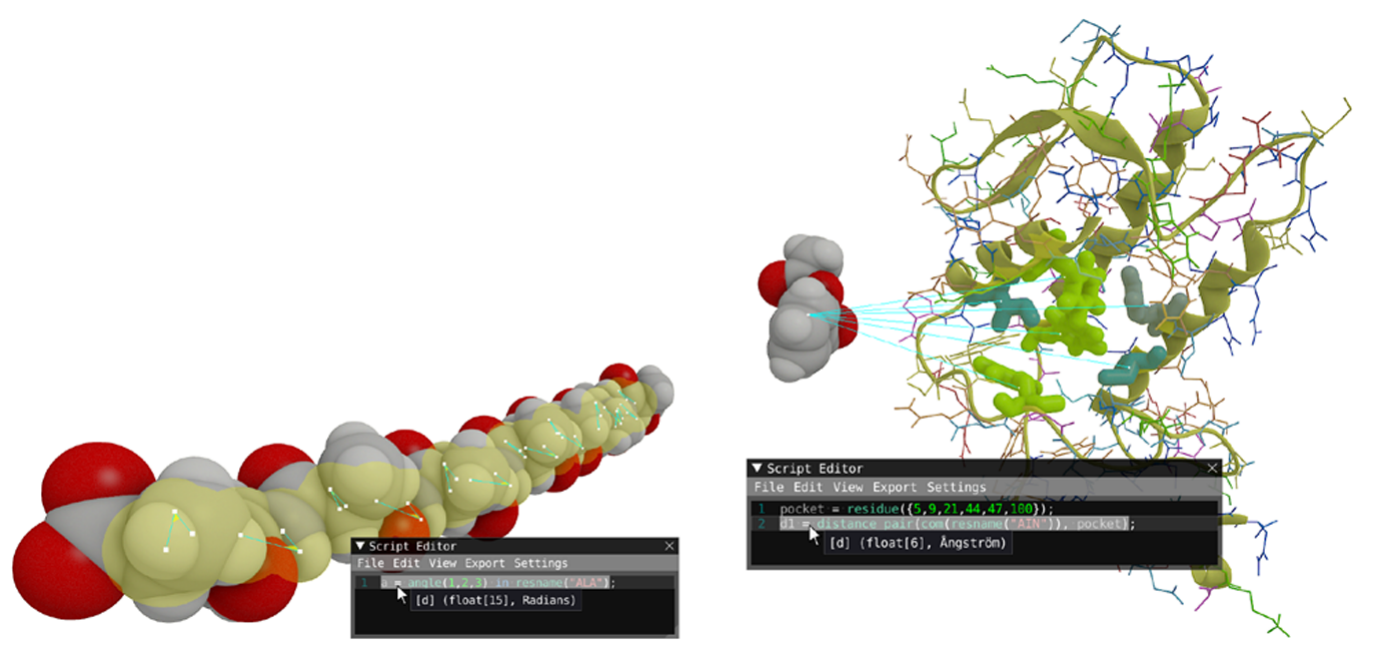
(a) Visualization of the angle formed by local atom indices within all residues named *ALA*. The involved atoms are highlighted; the spatial positions are marked by white points connected by lines, and a wedge is shown to emphasize the angle. The resulting type has a size of 15 and corresponds to the number of contexts (residues named ALA) in which the operation was performed. (b) Visualization of the property d1, the pairwise distance between the center of mass of the ligand (residue named *AIN*) and the user-defined pocket defined as a list of residues indices. The resulting type has a length of 6, corresponding to the individual distances from the ligand to each of the pocket’s residues.

The scripting editor, Figure 1d and Figure 3, is a text editor in which the user can write scripts containing identifiers and expressions. Input parameters of functions are validated against the loaded topology of the system, enabling errors and feedback, e.g., referring to residue names or atomic elements that are not present. Another type of feedback occurs when the user hovers over an expression or subexpression with the mouse cursor. A visualization operation is then performed to provide visual feedback on the operation and what parts of the system are involved. This is achieved by embedding geometrical primitives, such as points, lines, and triangles, and highlighting structures in the spatial view. Examples of this can be seen in Figure 1 and Figure 4. This geometrical embedding effectively ties the expressions to the spatial interpretation of the data, providing a feedback channel for asserting the user’s intent. When the user evaluates the script, the expressions are evaluated over the trajectory frames.

#### Property Import/Export

The scripting language is designed to be lightweight and expressive rather than exhaustive and complex. In cases where the set of exposed operations becomes the limiting factor, the property data can be exported to a tabular format. This functionality can be found in the script editor window under File → Export. Temporal and 1D distributions can be exported to .csv or .xvg files. 3D distributions can be exported as .cube files, which can also encode molecule structures. This is utilized in the case of spatial distribution functions, where the reference structure is exported as the molecular structure.

Data can also be imported into the script using the command *import*. It currently supports temporal data in tabular format in .csv, .xvg, and .edr (Gromacs energy file). The import command takes a path to a file and an optional filter argument to specify the fields or columns to import. If the path to the file is relative, it is assumed to be relative to the workspace if present, otherwise to the loaded trajectory.

### Temporal View (f)

**Figure 5:**
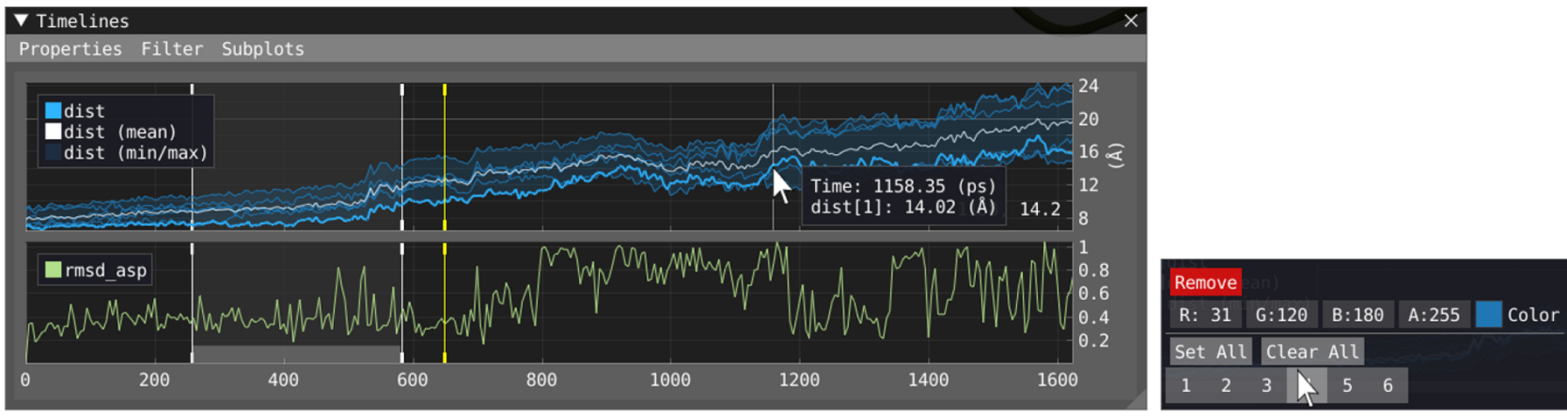
Temporal window (left) with two subplots showing two distinct properties evaluated through the script. The top subplot shows the individual lines within the population of *dist* as blue lines, with the population mean in white and population min/max as a transparent area. The bottom subplot shows a single line depicting property *rmsd*_*asp*. The currently viewed animation frame is shown as a vertical yellow line. The user hovers over a line corresponding to the first entry within the population of *dist*; therefore, its value is shown together with the time in a tooltip window. The context menu (right) is shown upon right-clicking a property’s legend entry, in which the visual representation of the property can be configured.

The temporal views, Figures 1f and 5, provide an overview of the timeline of the trajectory. The timeline shows the temporal evolution of properties as line plots. If the property consists of a population of values, the population min/max, mean, and variance can optionally be shown. Properties are placed within the subplot by dragging and dropping legend entries between the subplots or dragging properties from the Properties menu, which lists all available properties that can be shown in the temporal view. Hovering on properties (label or line) shown in the timeline window will provide visual feedback in the spatial view, similar to hovering the source expression in the script editor. If the property is currently shown in the distribution window, it will also be highlighted there.

#### Temporal Filtering

The temporal view also exposes an optional temporal filter, represented by a contiguous range (start and end time) shown as a shaded gray region in Figure 5 between vertical white lines. The temporal filter results in an extra evaluation pass for distribution and volume properties where only the frames in the range are considered. This enables the inspection of localized temporal trends within the data.

### Distribution View (h)

**Figure 6:**
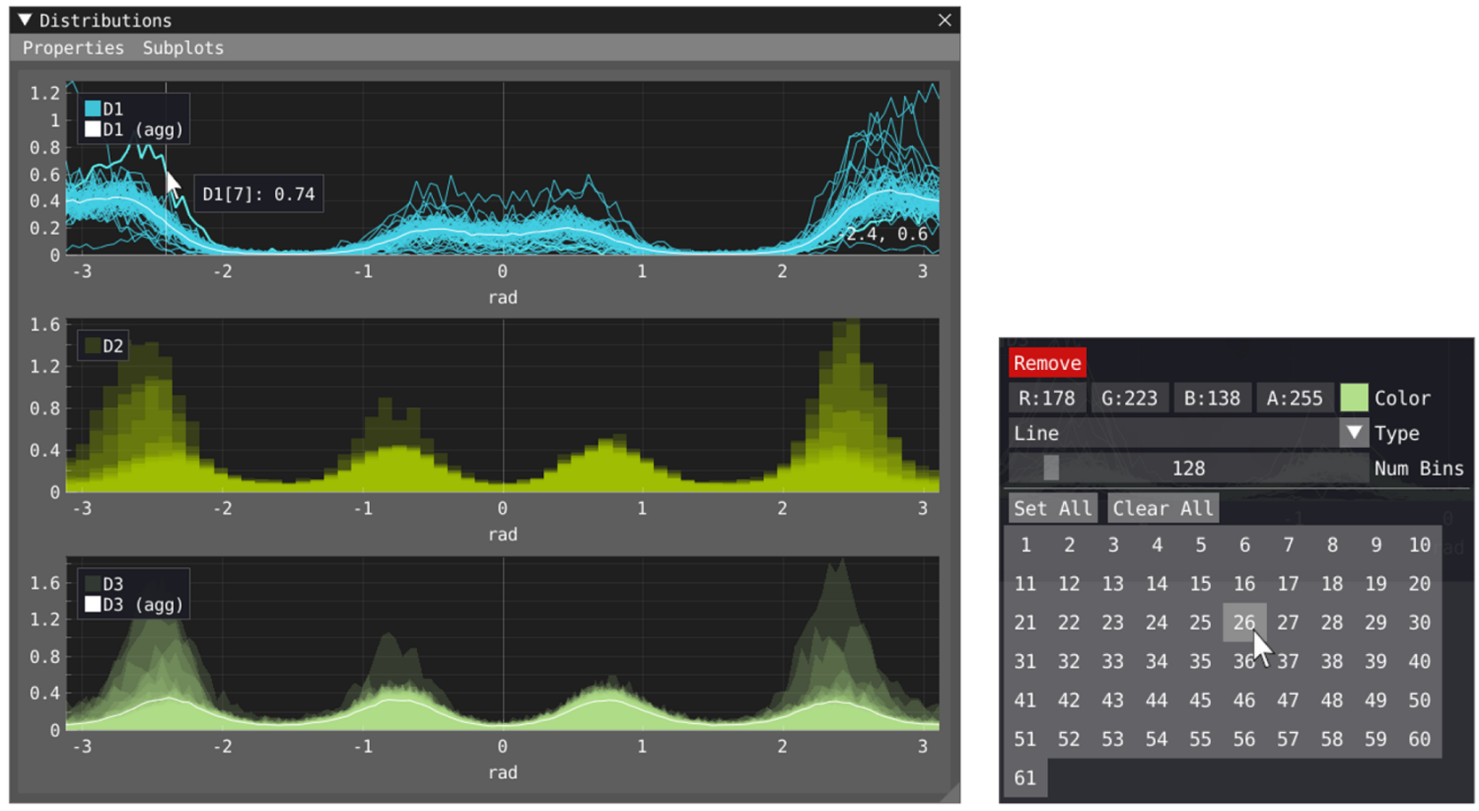
Distribution window (left) with three subplots showing three distinct properties: D1, D2, and D3. The top subplot shows the individual distributions within the population of D1 as light blue lines and their aggregate, D1(agg) in white. The middle subplot shows the distributions of D2 as bars, and the bottom subplot shows the population of D3 as shaded areas and its aggregate D3(agg) as a white line. The mouse hovers over one of the lines in the plot, and its label is shown together with its value. The context menu (right) is shown upon right-clicking a property’s legend entry, in which the visual representation of the property can be configured.

The distribution views, Figure 1h and Figure 6, show properties that evaluate to 1D distributions. The distributions are evaluated and stored in memory as high-resolution histograms, and when shown in the distribution window, a down-sampled version is used, configurable by the user (in powers of 2). The down-sampling scheme is conservative, ensuring an accurate representation of the underlying distribution without the need to re-evaluate the data when changing the visual number of bins. Hovering on properties (label or line) shown in the distribution window will provide visual feedback in the spatial view, similar to hovering the source expression in the script editor. If the property is currently shown in the temporal window, it will also be highlighted there.

The distribution view is divided into a number of subplots configurable by the user, and their contents are controlled by dragging and dropping legend entries listed under Properties in the menu or from other subplots. The visual representation of each property can be configured by right-clicking the property’s legend, which opens a context menu (Figure 6, right). If the property consists of a population, then the visibility of the individual components within the population can also be controlled.

### Volume View (g)

**Figure 7:**
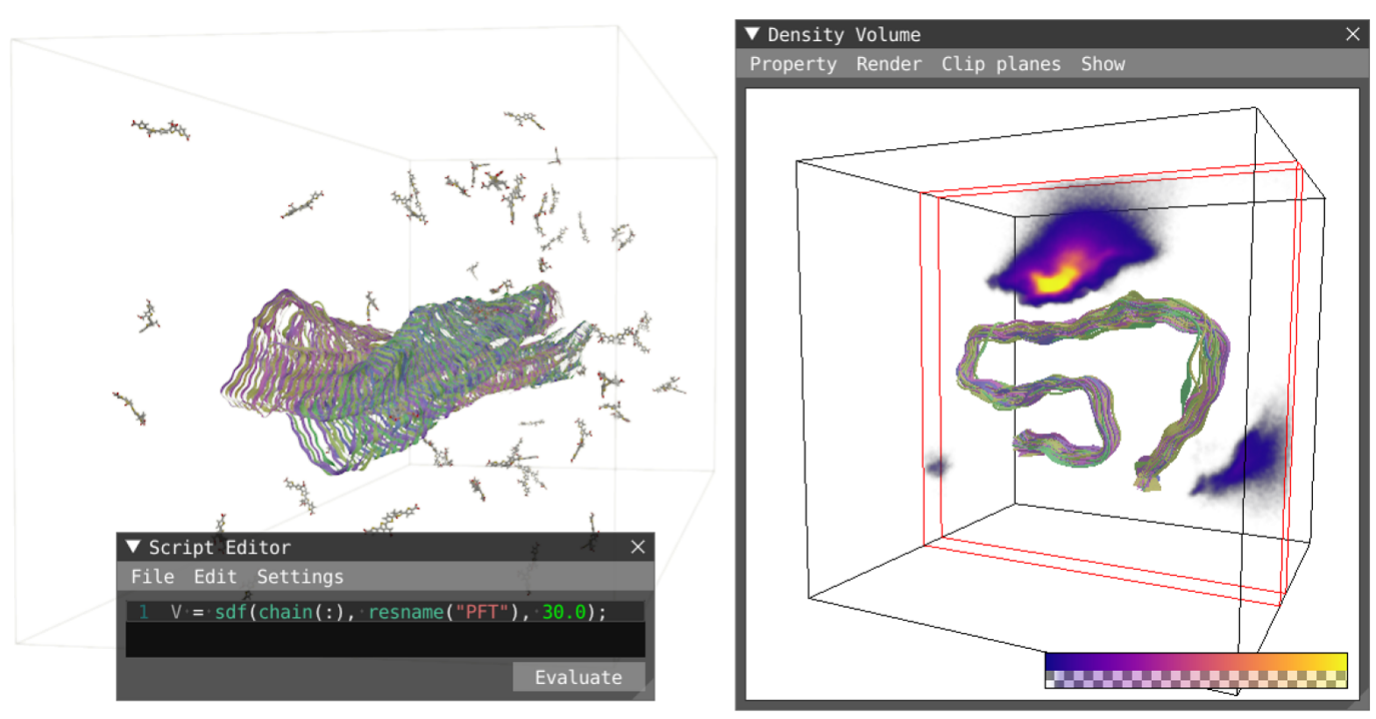
Volume view: the spatial density function of ligands labeled PFT in relation to stacked chains that form amyloid fibrils. Each chain serves as a reference frame in which the spatial occurrence of the ligands is aggregated and contributes to the resulting total density. Clip planes have been applied to provide a tight cross-section of the density close to the reference structures. The reference structures (chains) are shown in conjunction with the density volume in order to serve as a spatial reference.

The volume view, Figure 1g and Figure 7, shows properties that evaluate into volumes, e.g., Spatial Distribution Function, exposed in the script editor as sdf. Volumes can be rendered using direct volume ray-casting, where the density values are mapped to color and opacity using a predefined set of transfer functions or as user-defined iso-surfaces. The applied transfer function is optionally shown as a legend in the window for clarity.

Volume properties resulting from sdf also contain metadata that can optionally be shown within the volume window. In the case of sdf, the metadata are the reference structures used during the evaluation supplied as the first parameter. The reference structures can optionally be shown as representations in conjunction with the density volume, as shown in Figure 7.

### Shape Space View (i)

**Figure 8:**
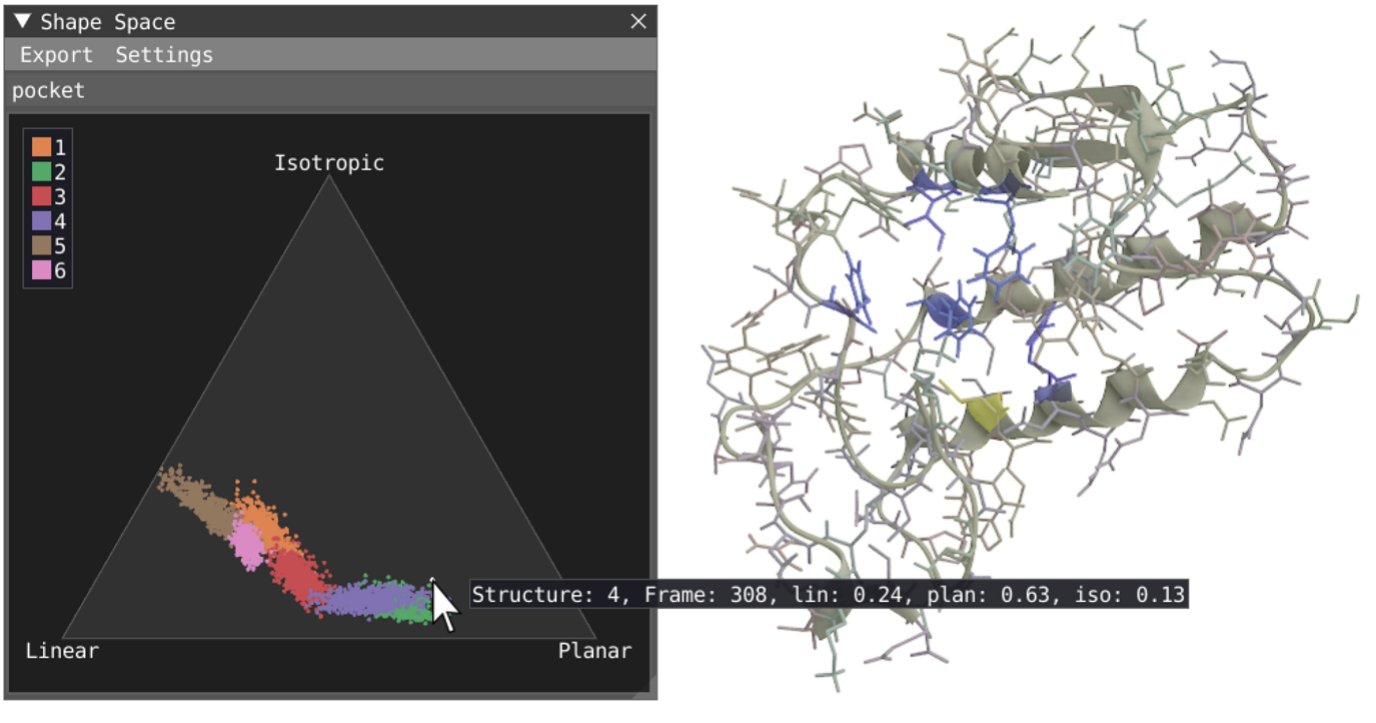
Shape space plot: a scatter plot of the temporal geometric anisotropy for a set of residues was accessed with the identifier pocket. Each color in the plot corresponds to one residue, and each point corresponds to a frame within the simulation. The three corners of the space correspond to the three anisotropic extremes: linear (lower-left), planar (bottom-right), and isotropic (top).

The shape space view, Figure 1i and Figure 8, serves as a complement to studying the geometrical deformation of structures over time. Temporally stable structures are required to derive stable reference frames. They are crucial for the temporal superpositioning of structures and a prerequisite for producing accurate results in operations such as spatial distribution function (SDF). The shape space is a temporal scatter plot where each point represents a structure configuration projected into a linear geometric anisotropic space spanned by three extremes, linear (line), bottom-left; planar (disc), bottom-right; and isotropic (spherical), top. By studying the spread of points within this space, it is possible to determine if a structure or set of structures undergoes substantial geometrical deformation over time and is thus unfit to serve as reference frames. In such cases, the user can further refine the structure selection by narrowing the selection to stable parts of the structures and excluding weakly connected extremities that degrade the stability of the reference frame. For details regarding the spatial distribution function and the shape space, see Skanberg et al.^20^

### Ramachandran Plot (e)

**Figure 9:**
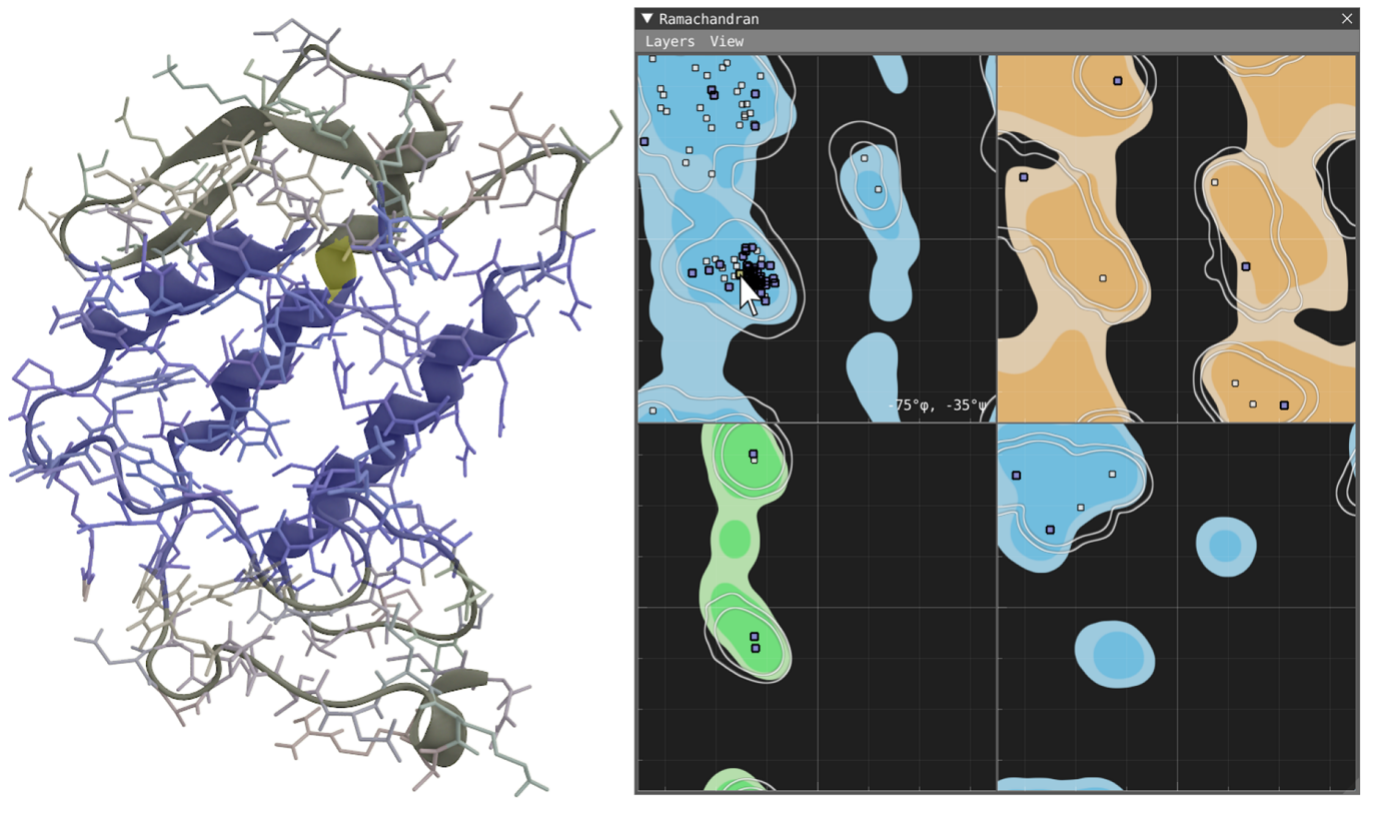
Ramachandran plot: the four views show the occurrence of backbone angles ϕ and ψ for residue classes: general (top left), glycine (top right), proline (bottom-left), and pre-proline (bottom-right). The background consists of configurable layers showing from bottom to top: reference distribution (derived from PDB top-500 proteins) was indicated as colored contour layers corresponding to fixed percentiles. Trajectory distribution (over full trajectory) as white isolines with matching percentiles. The top layer shows interactive points corresponding to the angles of the current configuration. The blue points show which residues are currently selected, and the yellow points show the point hovering with the mouse.

The Ramachandran plot,^21^Figure 1e and Figure 9, is a scatter plot where the coordinate axes correspond to dihedral angles ϕ and ψ from the backbone of protein structures. The plot is divided into four views corresponding to the peptide residue types: general, glycine, proline, and pre-proline. The coordinate axes of each plot are linked. The points represent proteins in their current configuration and are interactive, meaning the user can hover over and select them. The fill color of the points shows the current selection, where white means not selected, blue is selected, and yellow is highlighted (hovered). The views consist of configurable layers to provide context: The bottom-most layer represents a reference distribution derived from the PDB top 500 proteins as was originally suggested by Lovell et al.^22^ and is today considered common practice. The percentiles used for contour levels are the same as suggested by Lovell et al., 99.95% and 98% in the general case and 99.5% and 98% in the other cases. The next two layers represent distributions from the loaded data set, where one layer is the full trajectory and the other is the selected frame interval. Both distributions are scaled to match the contour levels of the reference distribution to enable A-to-B comparisons.

Each layer can be shown as filled contour levels or contour lines or mapped with a transfer function.

Lowell et al.^22^ employ a two-pass approach of normalized cosine smoothing in order to filter out points in specific percentiles and categorize the resulting regions as *allowed* and *favored*, where favored is more strict and contains fewer points than allowed. Partly, this technique was used to filter out outliers from the input data derived from Crystallography, which has an inherent uncertainty.

The goal is not to replicate the process as the points (backbone angles) now stem from simulated trajectories and do not suffer from the inherent uncertainty present in Crystallography and Cryo-EM. Thus, all points should be considered *equally*. Instead, we employ a normalized Gaussian kernel with a configurable standard deviation to spread the density. A Gaussian kernel was chosen since it is separable and well-suited for performance-critical applications.

## Workspace

The application’s state can be saved and loaded from workspace files with the default file extension .via. It contains representations, the script, stored selections, the current camera state, and general settings. The format is a text-based ASCII format and can be modified with any text editor. The only exception is stored selections, which are compressed and stored in Base64. In the Supporting Information, the reader can find an example workspace file in the online tutorials.

### Requirements, Performance, and Scalability

The software is designed to leverage thread-level parallelism by using an internal task pool, to which computation-intensive tasks are submitted. Examples of such tasks include script evaluations and Ramachandran and shape-space distributions. Each task may entail processing a range of elements (i.e., trajectory frames). In such cases, the range is split into subranges that are processed in parallel. The number of available worker threads in the thread pool is exposed as a user-configurable compile-time parameter in the CMake script. This ensures that the task system can scale to the number of hardware threads available. Note that one of the threads is always reserved as the application’s main thread for handling logic and rendering to maintain interactivity. The software is also written to utilize data-level parallelism through SIMD-instructions to accelerate the processing time of computation-intensive operations.

The application also employs a cache system for trajectory frames, which streams in and decompresses trajectory frames on demand. It has a user-configurable compile-time parameter in the CMake script that controls the available memory for the cache system.

#### System Requirements

The minimum requirement is a dual-core system with 4 GB of available system memory and a graphics accelerator supporting OpenGL 3.30. The recommendation is to have a multicore system (4+) and at least 8 GB of available system memory as the evaluation performance scales with the number of threads available.


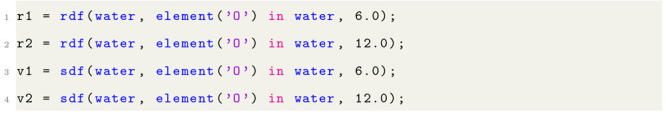
Listing 3: Properties r1 and r2 and v1 and v2 evaluate the radial distribution function of oxygen in water with respect to water and the spatial distribution of oxygen in water with respect to water in data set 2.


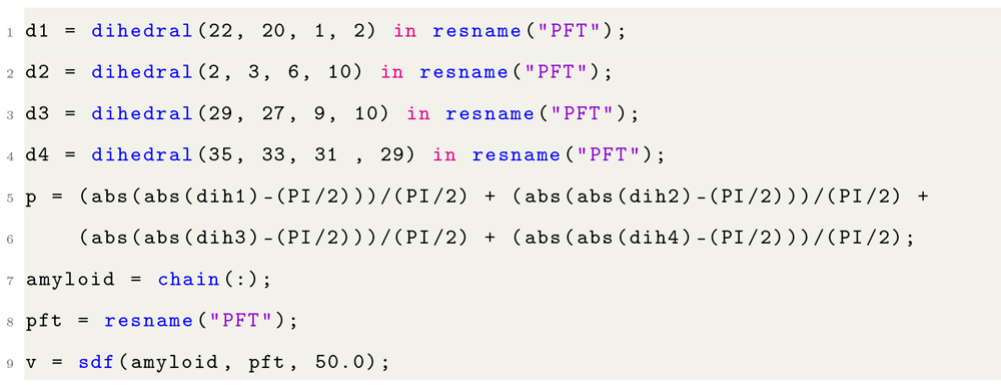
Listing 4: Properties p and v which represent the planarity and the spatial distribution of “PFT” molecules with respect to the chains in the system.

#### Performance Measurements

Tables 1 and 2 contain measurements of evaluations of specific properties (listings 3, 4) performed for two different data sets. The evaluated properties (with the exception of p) have been chosen as they represent both computation- and memory-intensive operations that access a substantial portion of each evaluated frame. The measurements have been performed on a system comprised of an AMD Ryzen 9 7950 × 3D 4.2 GHz CPU with 128 GB of DDR5 memory. The number of threads was varied to measure the scalability of the evaluation. Tables 1 and 2 list the evaluation times in seconds next to the scaling factor shown in parentheses. The scaling with respect to the number of threads seems to follow a near-linear trend, with the exception of p, whose speedup factor is linear only up until four threads. Since p is computationally inexpensive compared to the other properties, we hypothesize that a substantial portion of the measured time stems from the overhead of initializing and synchronizing evaluation tasks. Detailed instrumentation is required for further analysis.

**Table 1:** Evaluation Times in Seconds and Speedup Factor for Properties r1, r2, v1, and v2 in Listing 3^a^

# threads	1	2	4	8	16
r1	9.4 (1.0×)	4.8 (1.96×)	2.4 (3.91×)	1.3 (7.23×)	0.8 (11.75×)
r2	38.4 (1.0×)	19.3 (1.99×)	10.0 (3.84×)	5.1 (7.52×)	2.7 (14.22×)
v1	33.3 (1.0×)	16.8 (1.98×)	8.5 (3.92×)	4.7 (7.09×)	2.8 (11.89×)
v2	137.4 (1.0×)	69.6 (1.97×)	35.1 (3.91×)	19.2 (7.16×)	10.9 (12.60×)

a Evaluated for data set 2, comprised of 50 512 atoms spanning 600 frames with a total in-memory size of 348 MB. There are 16 433 water molecules present in the system.

**Table 2:** Evaluation Times in Seconds and Speedup Factor for Properties p and v in listing 4^a^

# Threads	1	2	4	8	16
*p* seconds (speedup)	0.4 (1.0x)	0.2 (2.0x)	0.1 (4.0x)	0.1 (4.0x)	0.2 (2.0x)
*v* seconds (speedup)	28.8 (1.0x)	15.8 (1.82x)	8.1 (3.55x)	4.2 (6.85x)	2.5 (11.52x)

a Evaluated for data set 3, comprised of 161 742 atoms spanning 2345 frames with a total in-memory size of 4.24 GB. There are 253 chains and 61 “PFT” molecules present in the system.

## Conclusions

To summarize, the VIAMD application presented in this article allows us to considerably improve the workflow of MD analysis by tightly coupling visualization, property computation, and plotting within the same application, allowing for the leveraging of synergies formed by this tight coupling. This optimization of the common MD-analysis workflow offers various benefits, including interactivity, the ability to declare derived properties from spatial selections directly, and the visualization of properties to aid the user in asserting the operation and structures involved. The VIAMD application is a promising solution for improving the efficiency and accuracy of MD analysis and has the potential to advance research in many scientific disciplines. We hope that VIAMD can be a tool for dissemination since more and more MD trajectories are available online in repositories, and there is an initiative for a search engine prototype to explore collected MD data.^23^ To reach a larger audience of computational chemists, we plan to extend the type of data that could be analyzed with VIAMD and develop new functionalities in the future.

## Availability and Documentation

The source code is freely available on https://github.com/scanberg/viamd under the MIT license. VIAMD is under active development; therefore, design and implementation aspects may be subject to change. Documentation is available on the wiki page of VIAMD https://github.com/scanberg/viamd/wiki with pages dedicated to the software’s visual, analysis, and language components. A series of tutorials is also proposed to encourage users to use VIAMD. The data sets used in this article to illustrate the functionalities of VIAMD are also available on the wiki.

## Data sets

### Data Set 1: Alanine Chain

The default data set is supplied with the application and automatically loaded upon start. It is a small data set of 500 frames with a single chain of 15 Alanine residues.

### Data Set 2: Aspirin and Protein

This data set is available to download from the provided link. It consists of an aspirin ligand being pulled from its specific binding to phospholipase A2. The dynamic was performed from the complex’s crystal structure (1OXR).^24^

### Data Set 3: Amyloid Fibril and PFTAA

This nonbiased molecular dynamic simulation illustrates the binding of a pentameric oligothiophene used to detect amyloid-β(1–42), responsible for Alzheimer’s disease.^25^

## Data Availability

There are also online resources providing documentation, tutorials, and the data sets used in the article at https://github.com/scanberg/viamd/wiki

## Supporting Information Available

Representation types, script types, script function glossary, limitations, example workspace (PDF)
