# Supplementary material for: VIAMD: a Software for Visual Interactive Analysis of Molecular Dynamics
Source: J Chem Inf Model. 2023 Nov 27;63(23):7382–91. doi: 10.1021/acs.jcim.3c01033 (PMC10716899; doi:10.1021/acs.jcim.3c01033)
Supplement: Supplementary file 1 — ci3c01033_si_001.pdf [file ci3c01033_si_001.pdf]

VIAMD SUPPORTING INFORMATION

Robin Skånberg, Ingrid Hotz, Anders Ynnerman and Mathieu Linares

October 13, 2023

1 Representations

1.1 Representation Types

- *SpaceFill* - Atoms are visualized as spheres with a radius defined by the VDW-radius of the atomic element.
- *Licorice* - Covalent bonds are visualized as capsules connecting atoms.
- *Ribbons* - The Protein Backbone splines are visualized using a rectangular profile producing flat ribbons.
- *Cartoon* - The Protein Backbone splines are visualized using a profile based on its secondary structure, small circular (coil), elliptical (helix), and rectangular (sheet).

There is another group of common visualization types, surface representations. These are not present in the current version of the application but are planned for a future version.

1.2 Color Mapping

- *Solid Color* - Assign a solid color.
- *CPK* - Assign colors by element type, using a predefined palette: Oxygen: Red, Carbon: Black/Gray, Nitrogen: Blue, Hydrogen: White, etc.
- *Atom Type/Label* - Assign colors by the atoms label, the color is determined by hashing the atom label yielding a pseudo-random color.
- *Atom Index* - Assign colors by atom index, the color is determined by hashing the unique atom index yielding a pseudo-random color.
- *Element Type* - Assign colors by element type, the color is determined by hashing the element type yielding a pseudo-random color.
- *Residue Index* - Assign colors by residue index, the color is determined by hashing the unique residue index, yielding a pseudo-random color.

- *Residue Id* - Assign colors by residue id, notice that the id is different than the index and is not guaranteed to be unique. The color is determined by hashing the id yielding a pseudo-random color.
- *Property (Experimental)* - Assign colors by evaluating a float property and map the resulting value into a color using a predefined set of color maps.

2 Scripting Language

2.1 Keywords

`=` Assignment

`<=` Comparison: Less than or equal

`>=` Comparison: Greater than or equal

`!=` Comparison: Not equal

`==` Comparison: Equal

`and` Bitwise and

`or` Bitwise or

`xor` Bitwise xor

`not` Bitwise not, inversion

`in` Contextual operation

`of` Reserved

`out` Reserved

2.2 Base types

The language exposes a set of base types that form the foundations of the language. Any base type can be composed into an array. In the language, element access into the array is performed via the square bracket operator `[]`. In this article, we also use the square bracket operator to specify the length of array types, e.g. `int[3]` corresponds to an integer array of length three. The empty bracket represents a *varying* length.

`int` Signed integer

`irange` Signed Integer range

`float` Float

`frange` Float range

`string` String of characters representing text

`bitfield` Set of bits that represent selections (individual atoms)

Predefined Composite Types The following types are distinct types derived from base types used to interface with the application and enable implicit conversions.

`volume -> float[N][N][N]` The volume represents a three-dimensional distribution (histogram) and uses a fixed resolution N, which can be adjusted at compile time, the default value is 128.

`distribution -> float[M]` The distribution represents a one-dimensional distribution (histogram) and uses a fixed resolution M, which can be adjusted at compile time, the default value is 1024.

`position -> float[3]` Position serves as a alias to float[3] to allow for implicit conversions to extract positions. The supported types as input for implicit conversion are: `int`, `irange`, `bitfield`, `float[3]`, where integers and iranges are interpreted as atom indices.

2.3 Function glossary

In this section, we present a list of the built-in functions. The entries should be read as follows: `function_name(operand_type / optional_operand_type, operand_type / optional_operand_type, ...) -> return_type`

Selection operations

`all (void) -> bitfield` Selects all atoms within the current context.

`name / type / label (string[]) -> bitfield` Selects atoms by matching label.

`element (int[] / irange[] / string[]) -> bitfield` Selects atoms with a specific element.

`atom (int[] / irange[]) -> bitfield` Selects atoms by index.

`protein (void) -> bitfield[]` Selects residues that are considered proteins (determined by resname).

`water (void) -> bitfield[]` Selects residues that are considered water (determined by atomic structure).

`ion (void) -> bitfield` Selects residues that are considered ions (determined from monatomic residues and elements).

`ring (void) -> bitfield[]` Selects cyclic structures within the system which form loops through covalent bonds and have a length of 3-6.

`resname (string[]) -> bitfield[]` Selects residues by matching name.

`resid (int[] / irange[]) -> bitfield[]` Selects residues by id.

`residue (int[] / irange[]) -> bitfield[]` Selects residues by index.

`residue (bitfield) -> bitfield[]` Extracts residues from selected atoms within a bitfield.

`chain (int[] / irange[] / string[] -> bitfield[]` Selects chains by index or id.

`chain (bitfield) -> bitfield[]` Extracts chains from selected atoms within a bitfield.

`x / y / z (frange) -> bitfield` Selects atoms with cartesian coordinate within supplied range.

`within (float / frange, position[]) -> bitfield` Selects atoms within a certain radial extent.

`flatten (bitfield[]) -> bitfield` Flattens an array of bitfields into a single bitfield by using bitwise or.

`residue (bitfield[]) -> bitfield[]` Grows selections of residues which have at least one bit set to encompass the entire residue.

`chain (bitfield[]) -> bitfield[]` Grows selections of chains that have at least one bit set to encompass the entire chain.

Compute operations

`distance (position[], position[]) -> float` Computes the distance between the center of mass of two structures.

`distance_min (position[], position[]) -> float` Computes the minimum distance between two sets of structures.

`distance_max (position[], position[]) -> float` Computes the maximum distance between two sets of structures.

`distance_pair (position[], position[]) -> float[]` Computes the pair-wise distance between two sets of structures.

`angle (position[], position[], position[]) -> float` Computes the angle between three sets of structures.

`dihedral (position[], position[], position[], position[] -> float` Computes the dihedral (torsion) angle between four sets of structures.

`rmsd (bitfield) -> float` Computes the root-mean-square deviation of atomic positions for a structure.

`rdf (position[], position[], float / frange) -> distribution` Computes the Radial Distribution Function (RDF) from sets of positions. The first operand is the reference positions, which serve as the centers of the distributions. The second operand is the target positions, which compute distances to the centers. The third operand is the cutoff or range.

`sdf (bitfield[], bitfield, float) -> volume` Computes the Spatial Distribution Function (SDF) for a set of structures. The first operand corresponds to the structures used as reference frames for the density volume; the second operand corresponds to target atoms used to populate the density volume. The third operand is the cutoff and sets the symmetrical extent of the density volume. If multiple structures are passed as reference structures (first operand), the supplied structures must be identical as the result is superimposed from all reference structures.

`com (position[]) -> float[3]` Computes the center of mass of a set of positions.

`plane (position[]) -> float[4]` Computes plane which optimally fits for a set of supplied positions.

Special operations

`import (string, void / int[] / irange[] / string[]) -> float[]` Imports tabular data from a file. The string encodes the path to the file relative to the current workspace file (if present) or the loaded trajectory. The second argument is optional; only fields (columns) with matching indices or labels are selected if present.

3 Limitations

The software has been developed to serve the needs of a rather narrow user base, with its current set of features and their limitations a testament to that. In this section, we list the current limitations and ideas on how to address them in the future.

File Formats The supported file formats have been focused on Gromacs and its ecosystem. In the future, we aim to add support for LAMMPS, NAMD, CHARMM, and AMBER. The currently supported file formats for topologies:

- *gro* - Gromacs (ASCII, any precision)
- *pdb* - Protein Databank (ASCII, reduced precision)
- *xyz/arc/xmol* - XYZ formats in variations (ASCII)

The currently supported file formats for trajectories:

- *xtc* - Gromacs (binary, compressed, full precision)
- *trr* - Gromacs (binary, full precision)
- *pdb* - Protein Databank, frame encoded via MODEL/TER (ASCII, reduced precision)
- *xyz/arc/xmol* - XYZ formats in variations (ASCII, any precision)

Supported file formats for script import:

- *edr* - Gromacs Energy (binary)
- *xvg* - Grace Tabular (ASCII)
- *csv* - Comma Separated Values (ASCII)

Supported file formats for script export:

- *xvg* - Grace Tabular (ASCII)
- *csv* - Comma Separated Values (ASCII)
- *cube* - Cube file, volume + structure (ASCII)

Script Functions The current set of exposed script functions is implemented in *md_script_functions*, a part of *mdlib*, the foundational library for MD operations developed in parallel to VIAMD. The script can call the functions in three ways: during validation, evaluation, and visualization. Validation is invoked during the static type-checking phase. This enables functions to check that their supplied arguments are valid in the context of the loaded dataset (topology). If not, the function can produce an error to inform the user. The function can also invoked during validation to determine the length of the return type if its type is of varying length. Evaluation is invoked when the function is expected to produce its result and write it to the supplied destination. Visualization is invoked when the user, for example, hovers over the function in the script editor. During visualization, the function can create geometric primitives and highlight structures within the system. The multitude of ways a function can be called creates a steep threshold for beginners to overcome when implementing new functionality. In the future, we plan to simplify the interface to script functions, lowering the threshold for new users to contribute.

Instruction Sets VIAMD is currently designed around the x86-64 instruction set with the possibility to leverage SIMD operations via SSE/AVX/AVX2/FMA extensions if available. AVX512 has not yet been included as its adoption and potential future among consumer-grade processors has been uncertain. VIAMD should also be supported on other instruction sets, such as ARM, but it is currently not tested. There is a plan to support ARM SIMD extensions via ARM Neon in the future.

Graphics Library VIAMD has been developed with OpenGL as its graphics library, specifically OpenGL version 3.30, to strike a good balance of hardware support and available features. This means the current version of VIAMD will not work on more recent Apple hardware since Apple has shifted to its own Graphics API Metal. There is a plan to transition to Vulkan and Metal as Graphics APIs in the future. There is also the emergence of WebGPU, a successor to WebGL, which could be highly relevant to support a web-based version of the application.

Filtered Evaluation The current version only supports a single filtered evaluation range in conjunction with the full evaluation. This prohibits direct comparison of distributions from different sub-ranges within the trajectory. In future versions, there is planned support for multiple user-defined evaluation ranges.

Reference Structures in Spatial Distribution Functions The reference structures are given as the first argument to the script function *sdf*. In the current version, the selected structures need to be identical, meaning the element of each index within the structure needs to match among all reference structures. For future versions, there is a plan to match according to a maximum common sub-graph criteria.

Volume Visualization In its current version, the application only supports inspecting a single volume at any given time. This limits the ability to perform A-to-B comparisons between volumes. In future versions, there is a plan for supporting simultaneous inspection of multiple volumes, side-by-side or superimposed.

4 Example of Script

Listing 1 displays a more advanced scripting scenario complete with comments to facilitate the motivation behind the declared properties. The analysis is performed for Dataset 3: Amyloid Fibril and PFTAA, available in the online resources https://github.com/scanberg/viamd/wiki/5.-Datasets.

```

#This script is an example of how the VIAMD language can be used to derive parameters
#for the trajectory analysis. The data set used is available here
#('https://github.com/scanberg/viamd/wiki/5.-Datasets#dataset-3').
#This data set is composed of probes in interaction with the AB amyloid fibrils

#Count number of molecules in function of time adsorbed
nb = count(residue(resname("PFT") and within(2.0,protein)));

#By placing yourself at the first step of the simulation and using the Selection Query:
#residue(resname("PFT") and within(2.0,protein)) and Apply, you can save your selection as ads
#by right clicking one of the highlighted molecules.;
ads = residue({10629, 10633, 10635, 10637, 10653, 10659, 10661, 10665 ,10678:10679 , 10684, 10686});

#You can then define a representation using the "ads" filter. After evaluation of your script.
#Check that all the molecules stay adsorbed during the full trajectory.

#In the same way, by going at the last frame you can save your selection as abs_end
#by right clicking one of the highlitghted molecules.;
ads_end = residue({10628:10629, 10633, 10635:10638, 10643, 10647, 10650, 10653:10654, 10656, 10659, 10661, 10663:10665, 10669:10671, 10678:10679, 10682, 10684, 10686});

#This is to select all the molecules not adsorbed at the end
not_ads = residue(not ads_end and not protein);

#You can then define a representation using the "not_ads" filter. After evaluation of your script.
#Check that the molecules are never adsorbed during the full trajectory.

#Calculation of the dihedral angles for the molecules adsorbed from the beginning
```

```
dih1_ads = dihedral(22, 20, 1, 2) in ads;
dih2_ads = dihedral(2, 3, 6, 10) in ads;
dih3_ads = dihedral(29, 27, 9, 10) in ads;
dih4_ads = dihedral(35, 33, 31 , 29) in ads;

#Merging the values for the cental dihedral angles of the molecules adsorbed
dih_center_ads={dih2_ads,dih3_ads};

#Calculation of the planarity parameters for the molecules adsorbed from the beginning.
#This parameter was defined in the following paper ("J. Phys. Chem. A 118 (2014), 9820")

p_ads = (abs(abs(dih1_ads)-(PI/2)))/(PI/2)
+ (abs(abs(dih2_ads)-(PI/2)))/(PI/2)
+ (abs(abs(dih3_ads)-(PI/2)))/(PI/2)
+ (abs(abs(dih4_ads)-(PI/2)))/(PI/2);

#Calculation of the dihedral angles for the molecules never adsorbed
dih1_not_ads = dihedral(22, 20, 1, 2) in not_ads;
dih2_not_ads = dihedral(2, 3, 6, 10) in not_ads;
dih3_not_ads = dihedral(29, 27, 9, 10) in not_ads;
dih4_not_ads = dihedral(35, 33, 31 , 29) in not_ads;

#Merging the values for the cental dihedral angles of the molecules never adsorbed
dih_center_not_ads={dih2_not_ads,dih3_not_ads};

#Calculation of the planarity parameters for the molecules never adsorbed
#This parameter was defined in the following paper ("J. Phys. Chem. A 118 (2014), 9820")

p_not_ads = (abs(abs(dih1_not_ads)-(PI/2)))/(PI/2)
+ (abs(abs(dih2_not_ads)-(PI/2)))/(PI/2)
+ (abs(abs(dih3_not_ads)-(PI/2)))/(PI/2)
+ (abs(abs(dih4_not_ads)-(PI/2)))/(PI/2);

#Spatial distribution function of PFTAA molecules around the amyloid
amyloid = chain(:);
pft = resname("PFT");
v= sdf(amyloid,pft,50.0);

#By using the Shape Space window, filter to display only the ads subgroup.
#You can see the shape space distribution for the 12 molecules in the group.
#By hovering on the caption, identify the five molecules that have the most
#narrow distribution with a cluster of point close to linear.
#You can activate and deactivate the plot for a specific residue by clicking on the caption.
#By hovering over the caption, the molecule gets highlighted in the 3D view.
```

```
#Using those tools and by hovering build a subgroup for those five
    molecules and call it super_ads.

super_ads = residue({10633,10637,10665,10678,10679});

#Calculate the dihedral angle and group the central dihedral angles
     for this super_ads subgroup.

dih1_super_ads = dihedral(22, 20, 1, 2) in super_ads;
dih2_super_ads = dihedral(2, 3, 6, 10) in super_ads;
dih3_super_ads = dihedral(29, 27, 9, 10) in super_ads;
dih4_super_ads = dihedral(35, 33, 31 , 29) in super_ads;

dih_center_super_ads={dih2_super_ads,dih3_super_ads};

#Calculate the planarity parameter for this subgroup.
p_super_ads = (abs(abs(dih1_super_ads)-(PI/2)))/(PI/2)
+ (abs(abs(dih2_super_ads)-(PI/2)))/(PI/2)
+ (abs(abs(dih3_super_ads)-(PI/2)))/(PI/2)
+ (abs(abs(dih4_super_ads)-(PI/2)))/(PI/2);
```

Listing 1: An example of a script with extensive comments to provide insights into the process behind the analysis
